# Predictors of complex PTSD: the role of trauma characteristics, dissociation, and comorbid psychopathology

**DOI:** 10.1186/s40479-022-00208-7

**Published:** 2023-01-05

**Authors:** E. Guzman Torres, A. Krause-Utz, M. Sack

**Affiliations:** 1 Department of Psychosomatic Medicine and Psychotherapy, Klinikum rechts der Isar, Technische Universität, Munich, Germany; 2 Department Clinical Psychology, Institute of Psychology, Universiteit Leiden, Leiden, the Netherlands

**Keywords:** CPTSD, Symptom severity, Trauma characteristics, Comorbid disorders, Dissociation

## Abstract

**Background:**

Complex Posttraumatic Stress Disorder (CPTSD) has previously been associated with earlier trauma onset, repeated interpersonal traumatization, more dissociation, and more comorbid psychopathology. However, it is still debated if the afore-mentioned risk factors are related to CPTSD diagnosis or rather indicative of a more severe form of post-traumatic distress. The aim of this study was to compare patients with a CPTSD diagnosis to those with PTSD in trauma characteristics (onset, chronicity, interpersonal nature, familiarity with perpetrator), dissociation, and psychiatric comorbidities, while accounting for symptom severity.

**Methods:**

In total, *N* = 81 patients with a trauma history (*n* = 43 with CPTSD; *n* = 37 with PTSD) underwent diagnostic interviews by trained clinicians and completed measures on CPTSD symptom severity, trauma characteristics, and dissociation (Screening for Complex PTSD; Dissociative Experience Scale Taxon).

**Results:**

Patients with CPTSD reported earlier onset of trauma, more trauma perpetrated by acquaintances or family members, and more comorbidities than those with PTSD, also when accounting for symptom severity. No group differences in chronicity and dissociation were found. Severity of CPTSD was associated with earlier onset, familiarity with perpetrator, more comorbid (affective) disorders, and dissociation in both diagnostic groups.

**Conclusion:**

Findings largely confirm earlier research, suggesting that CPTSD is associated with traumatic events that start earlier in life and are perpetrated by acquaintances. Focusing on transdiagnostic symptoms, such as dissociation, may help to detain symptom deterioration. Due to the small sample size, findings need to be interpreted with caution and further research is needed to replicate findings in larger samples. Future research should also elucidate possible working mechanisms besides dissociation, such as emotion dysregulation or negative self-image.

**Supplementary Information:**

The online version contains supplementary material available at 10.1186/s40479-022-00208-7.

## Introduction

The 11^th^ revision of the International Statistical Classification of Diseases (ICD-11) recognizes Complex Posttraumatic Stress Disorder (CPTSD) as a new concept, intended to cover psychological reactions to early, enduring interpersonal traumatization [1]. In ICD-11, CPTSD is listed as sibling diagnosis alongside ‘classical ‘ PTSD. Both constructs are idiosyncratically related, following a potentially traumatizing event (PTE). In addition to ‘classical ‘ PTSD features (intrusions, avoidance, and hyperarousal), CPTSD comprises three domains of ‘disturbances in self-organization’ (DSO), i.e., emotion dysregulation, negative self-perception, and interpersonal disturbances [1, 2]. Estimated prevalence rates are 1.5% for PTSD and 0.5% for CPTSD [3].

There is growing evidence that CPTSD is clinically and conceptually distinct from PTSD [4, 5] in that it detects a group of patients exposed to earlier, more long-lasting and invasive events of primarily interpersonal nature (e.g., severe abuse and neglect, intimate partner violence, rape, sex trafficking, war, refugee trauma) [5, 6]. In previous research, childhood trauma was associated with increased probabilities of CPTSD over PTSD diagnosis [7]. Moreover, patients with CPTSD showed greater functional impairment [5], including more psychiatric comorbidities [8]. In another study, patients with CPTSD reported significantly more dissociation than patients with PTSD and a group of traumatized individuals without diagnosis [8]. In a dimensional manner, CPTSD symptom severity was associated with earlier trauma onset, acquaintance with the perpetrator [9] and higher levels of dissociation [10, 11].

Yet, the validity and utility of the CPTSD diagnosis continues to be debated. It has been questioned whether the afore-mentioned risk factors are related to CPTSD as a disparate diagnosis or to a more severe form of post-traumatic distress [12–14]. Interpersonal trauma was associated with more severe and persistent `classical` PTSD symptoms [9, 10]. Long-lasting traumatization as compared to single-incident trauma predicted more severe symptoms across the entire PTSD spectrum [17]. Furthermore, PTSD symptom severity was related to more comorbidities, such as depression and substance abuse [10, 15], and more physiological impairment. The utility of CPTSD as distinct diagnostic entity has thus been doubted [16, 17].

The aim of the present study was to shed more light on these associations. Patients with CPTSD diagnosis versus PTSD diagnosis were compared in terms of trauma characteristics (onset, chronicity, interpersonal nature), comorbidities, and dissociation. Based on previous research, we hypothesized that CPTSD diagnosis and severity were associated with earlier trauma onset, chronicity (iterated versus single-incident trauma), traumatization by acquaintance or family member, more dissociation, and more psychiatric comorbidities. We further explored if symptom severity accounts for differences between diagnostic groups.

## Methods

### Participants and procedure

The study was conducted at the department of Psychosomatic Medicine and Psychotherapy of the University Hospital ‘Rechts der Isar’, Munich (Germany). Ethical approval was granted by the local Ethics committee (Bavarian Hospital Statute). Data collection took place as part of routine assessment at the outpatient trauma section of the department in 2016. Diagnostic interviews were conducted by licensed psychotherapists. Three sessions of 50 min were scheduled for each patient. Prior to their initial therapy sessions, patients further completed questionnaires, described below. Inclusion criteria were exposure to at least one PTE and diagnosis of PTSD or CPTSD according to ICD. Overall, *N* = 81 patients (*M*_*age*_ = 39.74, *SD*_*age*_ = 11.08) were eligible for the study, most of them (*n* = 65; 80.2%) were female. *N* = 37 patients were diagnosed with PTSD, *N* = 43 with CPTSD. The groups did not differ significantly in age, gender, family status, and education (see Table 1). Due to missing values, data of *n* = 14 had to be excluded for a subset of analyses, resulting in a subsample of *N* = 67 for this sub-analysis (see below).

**Table 1:** Demographics and Clinical Characteristics in patients with Post-Traumatic Stress Disorder (PTSD) and complex PTSD (CPTSD)

	PTSD (*n* = 37) Mean ± SD	CPTSD (*n* = 43) Mean ± SD	Group statistics
*Age* [years]	39.49 ± 11.92	40.37 ± 10.20	*F*_*(1,78)*_ = 0.13, *p* = .721, *η*_*p*_^*2*^ = *0.002*
*Gender*			
*Female*	*n* = 28	*n* = 36	*Χ*^*2*^_*(1)*_ = *.805, p* = .370
*Male*	*n* = 9	*n* = 7	
*Education level (German)*			
Volkshochschule (9 years)	*n* = 9	*n* = 11	*Chi*^*2*^_*(1)*_ = 2.56, *p* = .275
Realschule (10 years)	*n* = 9	*n* = 10	*Chi*^*2*^_*(1)*_ = 2.73, *p* = .256
Abitur (12 years)	*n* = 4	*n* = 4	*Chi*^*2*^_*(1)*_ = 2.72, *p* = .257
Higher education	*n* = 7	*n* = 17	*Chi*^*2*^_*(1)*_ = 5.38, *p* = .070
*Family status*			
*Single*	*n* = 16	*n* = 25	*Chi*^*2*^_*(2)*_ = 1.50, *p* = .472
*Married*	*n* = 13	*n* = 11	
*Divorced*	*n* = 5	*n* = 5	
*Previous psycho-therapeutical treatment*	*n* = 25	*n* = 38	*Chi*^*2*^_*(1)*_ = 0.74, *p* = .785

A power analysis indicated that a subsample of *N* = 67 was still sufficiently large to detect a medium to large effect size (*ƒ*^2^ = 0.26) with a ß-error probability of 0.05% (Power of 0.95).

## Measures

### Trauma characteristics and CPTSD symptom severity

The Screening for Complex PTSD (SkPTBS) was used to assess trauma characteristics and CPTSD severity [9, 18]. This scale was developed as a screening measure to facilitate diagnosis of CPTSD and to initiate appropriate treatment steps. The five items of the subscale ‘influential features and risk factors’ were used to assess trauma onset, chronicity, and acquaintance with perpetrator with respect to the index trauma (‘PTE’). Sixteen items of the subscale ‘CPTSD symptoms’ were used to measure CPTSD symptom severity. These items assess symptoms related to Disturbances of Self Organization, as proposed in ICD-11, i.e., disturbances in emotion regulation, negative self-concept, disturbances in relationships, and sexual and somatic dysfunctions. Items are scored between 0 (does not apply at all) to 6 (entirely). The scale showed good psychometric properties and clinical validity [9, 18]. Internal consistency was α = 0.82.

### Comorbid psychopathology

Unstructured clinical interviews based on ICD 10 criteria were administered to assess CPTSD, PTSD, and comorbid conditions, such as affective disorders, substance use disorders, somatoform disorders, or other anxiety disorders.

## Dissociation

The Dissociative Experiences Scale Taxon (DEST) a self-report scale with eight items (between 0% (not at all) and 100% (always)) was employed to assess dissociation [19]. Established cut-off for pathologic dissociation is 20. Internal consistency was α = 0.82.

### Statistical analyses

Statistical analyses were conducted using IBM SPSS Version 25. Trauma characteristics included `onset` (continuous: age in years), `chronicity` (dichotomous: single versus repetitive event); `nature of trauma` (dichotomous: interpersonal versus force-majeur), and `acquaintance with perpetrator` (coded as unknown perpetrator, acquainted perpetrator, or both). CPTSD symptom severity was represented by mean total scores of the SkPTBS subscale. Dissociation was operationalized as mean DEST scores. For the SkPTBS, data of *n* = 14 needed to be excluded. For the DEST, data from *n* = 2 participants had to be excluded, resulting in a final sample size of *N* = 67 and *N* = 81 for the separate analyses respectively.

To test the hypothesis, univariate or multivariate analyses of variance (ANOVA/MANOVA) or Chi^2^ tests were used to compare the CPTSD group to the PTSD group (*p* < 0.05, two-tailed). To investigate if afore-mentioned variables predicted CPTSD symptom severity irrespective of diagnosis, linear regression analysis or ANOVAs (for dichotomous/categorical variables) were performed. For the variable `acquaintance with perpetrator`, separate dummy variables were created and simultaneously included in the regression analysis. To investigate if group differences were driven by symptom severity, we repeated the above-mentioned analyses with the SkPTBS subscale `symptom severity` as covariate. Prior to the analyses, assumptions for regression analyses and collinearity diagnostics were checked. Normality assumption was met, heteroscedasticity-constant standard errors were applied. According to VIF and tolerance values, multicollinearity was unlikely.

## Results

### Group comparisons between CTPSD and PTSD

Results of the (M)ANOVAs and Chi^2^ tests are summarized in Table 2. Patients with CPTSD reported earlier age of trauma onset than those with a PTSD diagnosis. Moreover, patients with CPTSD reported more traumatization by an acquaintance or family member. Patients with CPTSD reported more overall comorbidities, especially affective disorders, than patients with PTSD. No significant group differences were found for chronicity or dissociation.

**Table 2:** Group comparisons between patients with Post-Traumatic Stress Disorder (PTSD) and patients with complex PTSD (CPTSD)

	PTSD (*n* = 37) Mean ± SD	CPTSD (*n* = 43) Mean ± SD	Group statistics
Trauma onset [age in years]	15.84 ± 12.77	9.41 ± 11.01	*F*_*(1,64,)*_ = 4.82, *p* = .032, *η*_*p*_^*2*^ = 0.07
Chronicity			
Single	*n* = 9	*n* = 9	*Chi*^*2*^_*(1)*_ = 0.27, *p* = .602
Multiple / Iterated	*n* = 21	*n* = 28	
Interpersonal acquainted			
Interp. unknown	*n* = 15	*n* = 30	*Chi*^*2*^_*(1)*_ = 7.88, *p* = .005
perpetrator	*n* = 10	*n* = 2	*Chi*^*2*^_*(1)*_ = 9.08, *p* = .003
Force-majeur, disease	*n* = 3	*n* = 3	*Chi*^*2*^_*(1)*_ = 0.83, *p* = .773
Dissociative symptoms			
DEST	18.57 ± 19.34	18.54 ± 16.94	*F*_*(df)*_ = 0.00, *p* = .999
*Comorbidities*			
Overall	*n* = 31	*n* = 42	*Chi*^*2*^_*(1)*_ = 4.81, *p* = .028
Affective disorders	*n* = 29	*n* = 42	*Chi*^*2*^_*(1)*_ = 7.42, *p* = .006
Anxiety disorders	*n* = 2	*n* = 1	*Chi*^*2*^_*(1)*_ = 0.523, *p* = .470
Substance use disorders	*n* = 1	*n* = 3	*Chi*^*2*^_*(1)*_ = 0.765, *p* = .386
Somatoform disorders	*n* = 7	*n* = 5	*Chi*^*2*^_*(1)*_ = 0.829,, *p* = .363

*CPTSD* Complex post-traumatic Stress Disorder, *DEST* Dissociative experiences scale taxon, *PTSD* Post-traumatic stress disorder

### Predictors of symptom severity

As summarized in Table 3., earlier age of trauma onset, higher dissociation, acquaintance with the perpetrator, and more comorbidities predicted higher CPTSD symptom severity. The effects of chronicity and interpersonal nature were insignificant.

**Table 3.**
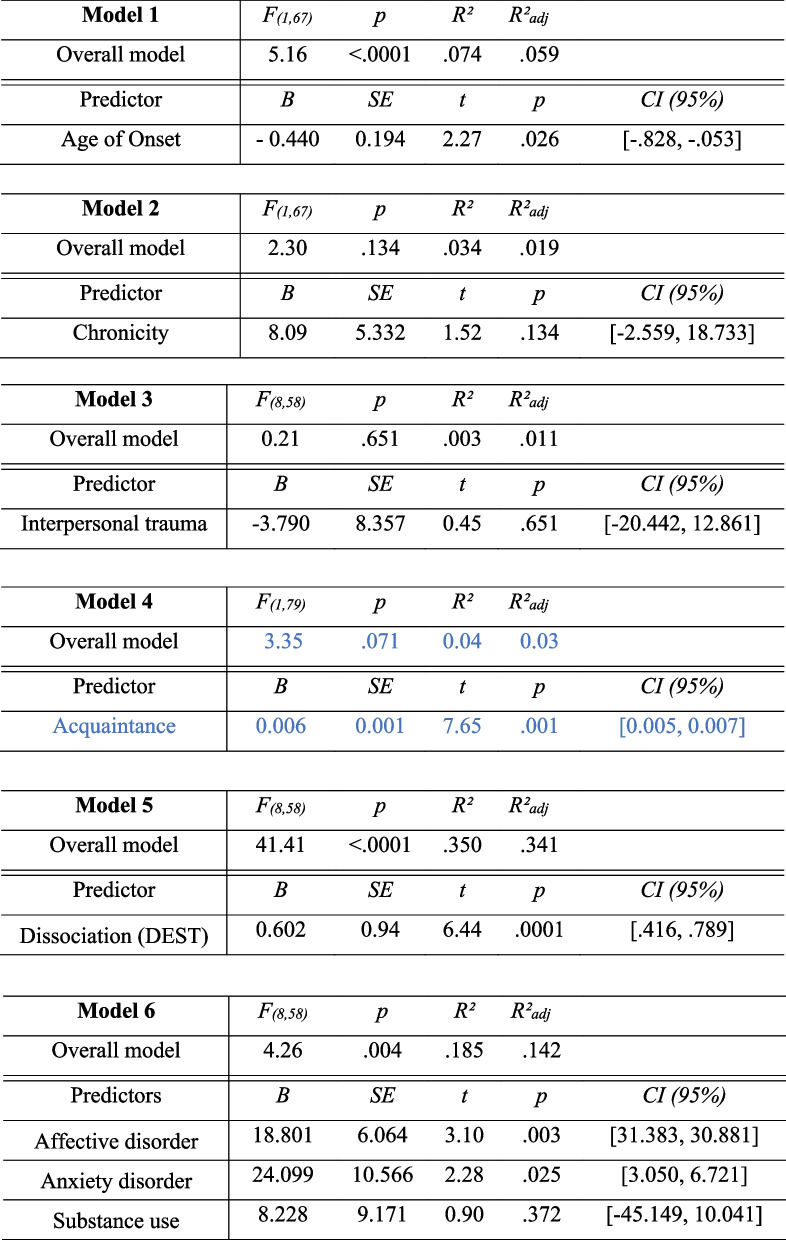
Linear regression analyses or univariate analyses of variance predicting CPTSD severity (SKPTBS total score) across the two patient groups

### Group differences controlling for symptom severity

When including the SkPTBS subscale score as covariate, group differences in trauma onset and interpersonal trauma remained significant, while group differences in comorbidities remained as a statistical trend (Supplemental Table 1).

## Discussion

The aim of this study was to investigate if specific trauma characteristics (age of onset, interpersonal nature, chronicity), dissociation, and comorbid psychopathology distinguish patients with a CPTSD versus PTSD diagnosis, while accounting for symptom severity.

CPTSD diagnosis was related to earlier trauma onset and familiarity with the perpetrator. These findings are in line with previous research, suggesting that CPTSD diagnosis detects a distinct group of patients exposed to earlier traumatic events of primarily interpersonal nature [5–7, 9]. Confirming previous research, we further detected more psychiatric comorbidities, especially affective disorders, in patients with CPTSD. These group differences could not fully be explained by higher symptom severity. We did not find group differences in chronicity, which may be explained by the relatively small sample size (i.e., low statisitical power) and the way it was operationalized (i.e., binary variable).

Interestingly, severity of dissociation was linked to CPTSD symptom severity across diagnostic groups. This suggests that dissociation is an important transdiagnostic mechanism that may increase symptom severity, e.g., by experiential avoidance or disrupted information processing [20, 21]. Monitoring dissociation and providing skills to regulate dissociative states may help to prevent symptom deterioration in traumatized individuals [21]. Future studies should investigate the role of other possible working mechanisms, such as emotion dysregulation and negative self-image.

By including a well-characterized sample of patients that received either a CPTSD or PTSD diagnosis in extensive diagnostic interviews, our study adds to the growing literature in this field. Findings need to be interpretated with caution due to several methodological limitations. Since the sample was relatively small, mostly female, and primarily included patients with similar cultural backgrounds, findings need to be corroborated in larger, more diverse samples. Moreover, certain subsamples or trauma types (war, refugee crisis, and torture etc.) may have been underrepresented.

Increasing knowledge on risk factors of CPTSD will have important clinical implications. Focusing on specific risk factors at early stages of the therapeutic progress may facilitate appropriate treatment choices. More research is needed to provide further evidence for the utility of CPTSD as a separate diagnostic entity. Ultimately, this will help to improve psychotherapeutic interventions for patients affected by complex traumatization.

## Conclusion

Overall, our study supports previous findings associating CPTSD with earlier trauma onset, more trauma perpetrated by acquaintances, and more comorbidities than those with PTSD diagnosis. Severity of CPTSD additionally correlated with more dissociation in both diagnostic groups. Given the small sample size, findings need to be interpreted with caution and more research is needed to elucidate whether CPTSD diagnosis detects a separate group of individuals or is rather part of a larger spectrum of trauma-related disorders. More research with larger samples is needed to replicate or extend findings and to elucidate possible working mechanisms aside from dissociation, such as emotion dysregulation or negative self-image.

## Supplementary Information

**Additional file1**: **Table 3** Results of the (m)ultivariate analyses of variance controlling for CPTSD symptom severity.

## Data Availability

According to European law (GDPR), data containing potentially identifying or sensitive patient information are restricted; our data involving clinical participants are not freely available in the manuscript, supplemental files, or in a public repository. Data access can be requested on reasonable demand via the corresponding author.
