# Supplementary material for: Predictors of complex PTSD: the role of trauma characteristics, dissociation, and comorbid psychopathology
Source: Borderline Personal Disord Emot Dysregul. 2023 Jan 5;10:1. doi: 10.1186/s40479-022-00208-7 (PMC9814348; doi:10.1186/s40479-022-00208-7)
Supplement: Supplementary file 1 — Additional file1: Table 3 Results of the (m)ultivariate analyses of variance controlling for CPTSD symptom severity. [file 40479_2022_208_MOESM1_ESM.docx]

Table 3

*Results of the (m)ultivariate analyses of variance controlling for CPTSD symptom severity*

|  |  |  |  |  |  |  |
| --- | --- | --- | --- | --- | --- | --- |
| Model 1 |  |  |  |  |  |  |
| Predictor | Outcome | *B* | *SE* | *t* | *p* | *CI (95%)* |
| Diagnosis | Age of onset * | 5.98 | 2.90 | 2.06 | .044 | [0.178, 11.784] |
| Symptom severity |  | -0.13 | 0.07 | 1.75 | .085 | [-0.278, 0.018] |
|  |  |  |  |  |  |  |
| Model 2 |  |  |  |  |  |  |
| Predictor | Outcome | *B* | *SE* | *Wald* | *p* | *CI (95%)* |
| Diagnosis | Chronicity | 0.14 | 0.60 | 0.06 | .810 | [-1.015, 1.435] |
| Symptom severity |  | 0.02 | 0.02 | 1.97 | .151 | [-0-015, 0.051] |
|  |  |  |  |  |  |  |
| Model 3 |  |  |  |  |  |  |
| Predictor | Outcome | *B* | *SE* | *Wald* | *p* | *CI (95%)* |
| Diagnosis | Interpersonal nature | 1.32 | 0.54 | 6.93 | .008 | [0.457, 2 173] |
| Symptom severity |  | 0.08 | 0.02 | 0.38 | .587 | [-0.024, 0.043] |
|  |  |  |  |  |  |  |
| Model 4 |  |  |  |  |  |  |
| Predictor | Outcome | *B* | *SE* | *t* | *p* | *CI (95%)* |
| Diagnosis | Dissociation | 6.39 | 3.51 | 1.82 | .074 | [-0.621, 13.356] |
| Symptom severity |  | 0.61 | 0.09 | 6.72 | <.0001 | [0.430, 0.792] |
|  |  |  |  |  |  |  |
| Model 5 |  |  |  |  |  |  |
| Predictor | Outcome | *B* | *SE* | *Wald* | *p* | *CI (95%)* |
| Diagnosis | Comorbidities | 1.71 | 10.35 | 2.18 | .067 | [-1.733, 19.917] |
| Symptom severity |  | .061 | 0.45 | 6.97 | .013 | [0.005, 1.548] |

Note: CPTSD Symptom severity was measured by the SKPTBS total score; * controlled for current age (B=0.16, SE=0.13, t=1.23, p=.224, CI: [-0.102, 0.425]).

|  |
| --- |
